# Community-based directly observed therapy (DOT) versus clinic DOT for tuberculosis: a systematic review and meta-analysis of comparative effectiveness

**DOI:** 10.1186/s12879-015-0945-5

**Published:** 2015-05-08

**Authors:** Cameron M Wright, Lenna Westerkamp, Sarah Korver, Claudia C Dobler

**Affiliations:** Division of Pharmacy, School of Medicine, University of Tasmania, Hobart, TAS Australia; Spectrum Health, Grand Rapids, MI USA; National Tuberculosis Centre, Thimi, Nepal; University of Western Sydney, Sydney, NSW Australia; NHMRC Centre of Research Excellence in Tuberculosis Control, University of Sydney, Sydney, NSW Australia

**Keywords:** Tuberculosis, Directly observed therapy, DOT, Community-based DOT, Clinic DOT, Adherence

## Abstract

**Background:**

Directly observed therapy (DOT), as recommended by the World Health Organization, is used in many countries to deliver tuberculosis (TB) treatment. The effectiveness of community-based (CB DOT) versus clinic DOT has not been adequately assessed to date. We compared TB treatment outcomes of CB DOT (delivered by community health workers or community volunteers), with those achieved through conventional clinic DOT.

**Methods:**

We performed a systematic review and meta-analysis of studies before 9 July 2014 comparing treatment outcomes of CB DOT and clinic DOT. The primary outcome was treatment success; the secondary outcome was loss to follow-up.

**Results:**

Eight studies were included comparing CB DOT to clinic DOT, one a randomised controlled trial. CB DOT outperformed clinic DOT treatment success (pooled odds ratio (OR) of 1.54, 95% confidence interval (CI) 1.01 – 2.36, p = 0.046, I^2^ heterogeneity 84%). No statistically significant difference was found between the two DOT modalities for loss to follow-up (pooled OR 0.86, 95% CI 0.48 to 1.55, p = 0.62, I^2^ 83%).

**Conclusions:**

Based on this systematic review, CB DOT has a higher treatment success compared to clinic DOT. However, as only one study was a randomised controlled trial, the findings have to be interpreted with caution.

**Electronic supplementary material:**

The online version of this article (doi:10.1186/s12879-015-0945-5) contains supplementary material, which is available to authorized users.

## Background

Tuberculosis (TB) is a major cause of disease and death worldwide. In 2012, an estimated 8.6 million people around the world developed active TB and 1.3 million people died of the disease [1]. One of the challenges for effective TB control is ensuring patients with TB complete a full course of treatment [2].

With directly observed therapy (DOT) - a TB control strategy recommended by the World Health Organization (WHO) - patients are observed as they take their medicine, with the aim to increase adherence to treatment [3]. Supervision of TB treatment through DOT at health clinics is resource-intensive and can put additional pressure on health systems, particularly in settings with a high TB burden and constrained resources [4]. It can also be inconvenient for patients to visit a health clinic daily for treatment supervision, for reasons including travel and waiting times [5]. DOT also adds direct (e.g. transport costs) and indirect costs (e.g. in terms of time lost) to treatment [6] and these have been shown in several cases to be higher per case successfully treated for clinic DOT versus community-based DOT (CB DOT) [6-8]. For these reasons, CB DOT presents an alternative for policy makers, especially when the DOT provider is acceptable and accessible to the patient [5]. As a 2003 WHO report on community contribution to TB care pointed out, “Organised community groups, peer groups, chosen members of the community, and family members all have the potential to act as supervisors to ensure completion of treatment and hence cure” ([4], p. 24).

A systematic review and meta-analysis published in 2009 assessed 34 studies (randomised controlled trials and observational studies) that analysed 24 TB programmes using CB DOT [9]. The review found that programmes that offered a financial reward to supervisors in the community tended to have higher rates of treatment completion than those that did not. However, this result may have been due to chance (85.7% versus 77.6% respectively, p = 0.15). The focus of this review was on describing characteristics of CB DOT programmes (analysed based on patient characteristics, operational or organisational factors) and their impact on the treatment outcomes of CB DOT. However, treatment outcomes for CB DOT were not compared with conventional clinic DOT.

Family members have been shown in some settings to be a possible option for delivering CB DOT [10-15]. However, Frieden and Sbarbaro have questioned the appropriateness of routine family DOT and the extent to which these results can be generalised [16]. From a programmatic point of view, community health workers (CHWs) or community volunteers (CVs) may represent a group that can be more systematically trained, motivated and monitored than family members.

A Cochrane review, last updated in 2007, assessed the effectiveness of DOT compared to self-administered treatment (SAT) [17]. It also looked at the relative efficacy of different types of DOT delivery. It found a small benefit from CB DOT supervised at home (with providers including family members, CVs and CHWs), compared to SAT, in terms of treatment success (risk ratio (RR) 1.09, 95% CI 1.02-1.16; 1,365 participants, 3 trials [12,13,18]). There was no benefit of clinic DOT compared to SAT (RR 0.92, 95% CI 0.78-1.08, 444 participants, 2 trials [12,19]). There was only one randomised controlled trial (RCT) that compared treatment success between DOT delivered by a family member and clinic DOT [14], and one RCT that compared cure rate between CHW-supervised DOT and clinic DOT [20]. Both studies showed no significant difference between the different DOT strategies.

Given the potential value of CB DOT as a means of treatment supervision, especially in resource-constrained settings, a systematic review assessing the effectiveness of CB DOT compared to conventional clinic DOT including all available studies is indicated to inform policies on DOT delivery.

This systematic review aimed to determine the effectiveness of CB DOT delivered by CHWs or CVs, in terms of treatment success and loss to follow-up, compared to clinic DOT.

## Methods

### Definitions

DOT was defined as a treatment strategy where “an appointed agent directly monitors people swallowing their anti-TB drugs” [17]. CB DOT was defined as DOT provided in the community by CHWs or CVs; clinic DOT was defined as DOT delivered at a fixed health clinic, usually by a government health worker.

The definitions for CHWs and CVs in this review were based on the Global Tuberculosis Report 2013 ([1], p. 35):

CHWs were defined as “people with some formal education who have been given training to contribute to community-based health services, including TB prevention and patient care and support. Their profile, roles and responsibilities vary greatly among countries, and their time is often compensated by incentives in kind or in cash.”

CVs were defined as “community members who have been systematically sensitized about TB prevention and care, either through a short, specific training scheme, or through repeated, regular contact sessions with professional health workers.”

We included studies where the role of the persons delivering CB DOT was consistent with the definitions above for CHW or CV, independent of the term used in the study. Family members providing DOT for their relatives were not included in this definition of CHW or CV.

Treatment success was defined as the sum of cured patients and patients with completed treatment, using WHO definitions [21]. Loss to follow-up was defined as “a TB patient who did not start treatment or whose treatment was interrupted for two consecutive months or more” ([21], p. 6). In the absence of reporting of the numbers lost to follow-up, the reported number defaulting was used instead.

### Search strategy and data extraction

The Preferred Reporting Items for Systematic Reviews and Meta-Analyses (PRISMA) statement was used to guide the methodology and reporting of this systematic review and meta-analysis [22]. We searched the following literature databases: MEDLINE through OvidSP and through PubMed (1950 to 9 July 2014), EMBASE through OvidSP (1980 to 9 July 2014) and PreMEDLINE (on 9 July 2014). Search terms included: ‘community networks’, ‘community health services’, ‘community health nursing’, ‘health services for the aged’, ‘community care’, ‘home care services’, ‘directly observed therapy’, ‘DOT’, ‘delivery of health care’, ‘community-based participatory research’ and/or ‘tuberculosis [drug therapy, therapy]’. The search strategy is detailed in Additional file 1.

Two authors (CMW and LW) independently reviewed studies for compliance with the selection criteria. Papers that passed the screening of title and abstract underwent full article review. The reference lists of the articles selected for final inclusion were also checked along with directly relevant review articles identified. The authors independently extracted study data including: 1) study objective(s); 2) study setting; 3) study design and population size; 4) incentives provided to supervisors; 5) inclusion and exclusion criteria for different DOT strategies; 6) comparator group(s); 7) study outcomes & results; 8) study limitations; 9) main conclusion(s) and 10) study funding source(s). Disagreements in article selection and data extraction were settled through discussion.

### Study inclusion criteria

Studies were included if they reported TB treatment outcomes for CB DOT compared to clinic DOT. There was no restriction on the type of TB (pulmonary, extra-pulmonary, drug-sensitive or drug-resistant) or method of diagnosis (microbiological or radiological with typical clinical presentation). We included RCTs and non-randomised studies (interventional and observational). Studies were included independent of data analysis method (intention-to-treat or other). CB DOT and clinic DOT had to be given for the same time period within a study and for a minimum of the intensive phase of treatment to be included.

### Exclusion criteria

The following studies were excluded from the review:

1. Studies without a full-text version available in English.
2. Letters to the editor, notes, comments, news or newsletter items, doctoral theses, conference proceedings (not published in a peer-reviewed journal) and meeting minutes.
3. Studies that did not involve CHWs or CVs delivering CB DOT, for at least the intensive phase of treatment. For example, studies utilising family members only as DOT supervisors were excluded.
4. Studies in which clinic DOT, for at least the intensive phase of treatment, was not a comparator group.
5. Studies in which CB DOT and clinic DOT were not directly comparable. This was either because CB DOT and clinic DOT treatment outcomes were not separated (e.g. in cases where family members and CHWs were both considered as CB DOT providers without separate results reported for each), or in cases where CB DOT was part of a comprehensive strategy incorporating increased TB awareness and training, and where this was not applied to the clinic DOT comparator.
6. Studies with a major selection bias evident in treatment allocation to CB DOT or clinic DOT. This included:
  - studies in which patients were allocated to CB DOT because they were not compliant with clinic DOT or had refused to participate in clinic DOT;
  - non-randomised studies in which clinic DOT and CB DOT were delivered to patients living in different geographical locations and where these locations were not matched on population size, level of economic activity or accessibility of health services – for example, patients from rural locations or living far from a health centre allocated to one type of DOT, with patients from an urban area or living near to a health centre allocated to the other, and
  - non-randomised studies in which patient characteristics (e.g. socio-economic status) or severity of TB disease influenced the allocation of DOT type.

Poor study quality for other reasons than selection bias was not an exclusion criterion.

### Outcomes and analysis

Data from included studies were analysed for the CB DOT and the clinic DOT intervention. If studies additionally included patients undergoing other types of DOT supervision (e.g. DOT provided by private physicians) or SAT, we only included the patients receiving CB DOT or clinic DOT.

The primary outcome was the proportion of patients who were successfully treated; the secondary outcome was the proportion of patients who were lost to follow-up. Meta-analyses were performed for both outcomes.

Review Manager Version 5.3 (Nordic Cochrane Centre, Copenhagen, Denmark) was used to perform the meta-analyses. As the studies were from different countries and in different permutations of CB DOT and clinic DOT, we expected heterogeneity beyond that explained by random differences between patient groups in the individual studies. Thus, a random effects meta-analysis for the data was deemed more appropriate than assuming fixed variation. Confidence intervals (CIs) were set at 95% and data were presented using odds ratios (ORs) calculated via the Mantel-Haenszel method. Heterogeneity was quantified using the I^2^ value described by Higgins and colleagues [23]. An I^2^ of 0% indicates no study heterogeneity, whilst progressively higher values represent greater inter-study heterogeneity.

### Assessment of study quality and risk of bias

Studies were assessed for quality using the Grading of Recommendations Assessment, Development and Evaluation (GRADE) criteria [24]. For RCTs, risk of bias was assessed using guidelines provided by the Cochrane Collaboration and those parts of the guidelines that were relevant were also used to conduct a risk of bias assessment for the included non-randomised studies [25].

## Results

### Study selection

**Figure 1:**
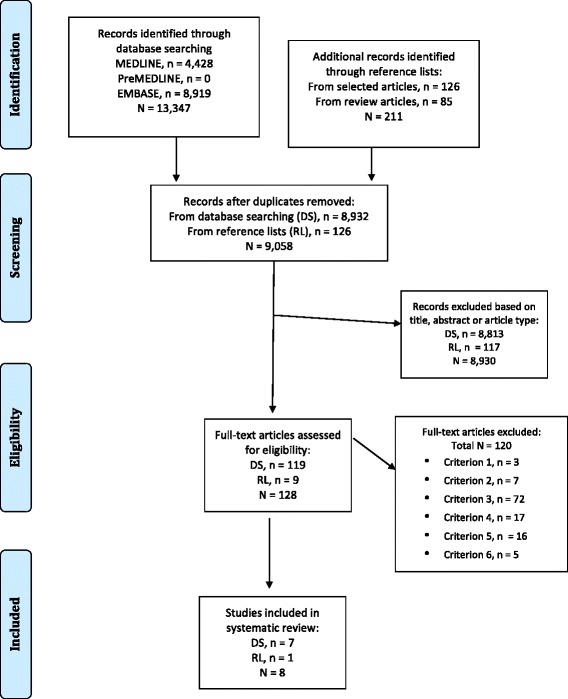
Flow diagram of study selection.

The literature search yielded 4,428 MEDLINE, 8,919 EMBASE and zero PreMEDLINE citations. In total, 8,932 publications were identified once duplicates were removed. Of these, 8,813 were excluded based on review of the title and abstract, leaving 119 to undergo full text review. Of these, seven studies fulfilled the eligibility criteria [13,20,26-30] and one further eligible study was identified from the reference list of one of the included studies [31]. Figure 1 shows the search strategy results in detail.

References of the directly related reviews identified through the search strategy yielded no additional eligible studies [9,17,32].

The studies rejected excluded under criteria five and six are included in Additional file 2 as each of these studies did compare CB DOT with clinic DOT, but not in a way permitting inclusion in this systematic review [12,14,18,33-50].

### Study characteristics

Of the eight studies included in the review, seven were non-randomised studies [13,26-31], and one was a RCT [20]. One study randomised patients to either DOT or SAT, however patients in the DOT arm were allowed to select their treatment supervisor (clinic, CV or family-supervised DOT) [13]. This study was therefore classified as a non-randomised trial based on the part that was relevant for this review [13]. It was also the only study where family DOT was an option. Of the other non-randomised studies, one was a prospective cohort study [26], two were non-randomised trials [27,28] and three were retrospective cohort studies [29-31].

Four of the studies focused on urban populations [27-30], two on rural populations [20,31] and two studies on mixed urban/rural populations [13,26]. India was the most represented country with three studies [29-31] and a further one study each was from South Africa [26], Thailand [13], Zambia [27], Tanzania [20] and Iraq [28].

The selected studies included 8,203 patients in total with 1,957 patients receiving CB DOT and 2,719 patients receiving clinic DOT. The remainder was either supervised through other means (e.g. DOT provided by a family member), received SAT rather than DOT, or was not assessed for treatment outcomes in the study (see Table 1). Of 4,676 eligible patients, 3,996 patients had outcomes of treatment success (primary outcome) or loss to follow-up (secondary outcome). The study from South Africa did not provide specific loss to follow-up data for the CB DOT and clinic DOT groups, thus patients lost to follow-up in this study were not included in the total 3,996 patients with the primary or secondary outcome assessed in the meta-analyses [26].

**Table 1:** **Summary of included studies**

**Authors and year of publication**	**Study location**	**Study design**	**Number of patients**	**Results**
Kamolratanakul *et al.* 1999 [13]	Thailand, mixed urban/rural	RCT (DOT versus SAT) - for DOT arm supervisor self-selected.	837 total in study. 415 randomised to DOT (provider type known for 410; 1 other did not receive DOT as allocated, 352 received DOT via family member), 422 randomised to SAT.	**Treatment success:**
				CB DOT: 27 of 34 (79%)
				Clinic DOT: 21 of 24 (88%)
			**58 included in meta-analysis.**	**Loss to follow-up:**
				CB DOT: 5 of 34 (15%)
				Clinic DOT: 1 of 24 (4%)
Kironde and Meintjies 2002 [26]	South Africa, mixed urban/rural	Prospective cohort study	769 total in study 50 transferred away from area and not included. 598 new patients (93 of these received SAT) and 121 retreatment patients (not included).	**Treatment success:**
				CB DOT: 164 of 228 (72%)
				Clinic DOT: 189 of 277 (68%)
			**505 included in meta-analysis**	**Loss to follow-up:**
				18.7% reported for the study overall but not broken down according to provider type.
Lwilla *et al.* 2003 [20]	Tanzania, rural	Open cluster RCT	**522 total in study and all included in meta-analysis.**	**Treatment success:**
				CB DOT: 117 of 221 (53%)
				Clinic DOT: 148 of 301 (49%)
				**Loss to follow-up:**
				CB DOT: 88 of 221 (40%).
				Clinic DOT: 74 of 301 (25%).
Miti *et al.* 2003 [27]	Zambia, urban	Non-randomised trial	**168 total in study and all included in meta-analysis**	**Treatment success:**
				CB DOT: 44 of 72 (61%)
				Clinic DOT: 47 of 96 (49%)
				**Loss to follow-up:**
				CB DOT: 6 of 72 (8%)
				Clinic DOT: 22 of 96 (23%)
Niazi and Al-Delaimi 2003 [28]	Iraq, urban	Non-randomised trial (sequential allocation to one treatment arm or the other)	**172 total in study and all included in meta-analysis**	**Treatment success:**
				CB DOT: 72 of 86 (84%)
				Clinic DOT: 59 of 86 (69%)
				**Loss to follow-up:**
				CB DOT: 10 of 86 (12%)
				Clinic DOT: 9 of 86 (10%)
Nirupa *et al.* 2005 [31]	India, rural	Retrospective cohort study	3019 total in study	**Treatment success:**
			2661 (88%) could be contacted for the study. Treatment results for only new sputum positive TB patients, N = 1131. 28 patients received SAT. Outreach workers (N = 238) excluded as neither CB DOT nor clinic DOT.	CB DOT: 526 of 666 (79%)
				Clinic DOT: 147 of 199 (74%)
			**865 included in meta-analysis**	**Loss to follow-up:**
				CB DOT 92 of 666 (14%)
				Clinic DOT: 34 of 199 (17%)
Singh *et al.* 2004 [29]	India, urban	Retrospective cohort study	**617 total in study and all included in meta-analysis**	**Treatment success:**
				CB DOT: 110 of 141 (78%)
				Clinic DOT: 367 of 476 (77%)
				**Loss to follow-up:**
				CB DOT: 21 of 141 (15%)
				Clinic DOT: 69 of 476 (14%)
Tripathy *et al.* 2013 [30]	India, urban	Retrospective cohort study	2099 total in study	**Treatment success:**
			Treatment cards of 1864 (89%) available for evaluation. Patients supervised by physicians (N = 95) removed from CB DOT results.	CB DOT: 475 of 509 (93%)
				Clinic DOT: 951 of 1260 (75%)
			**1769 included in meta-analysis**	**Loss to follow-up:**
				CB DOT: 13 of 509 (3%)
				Clinic DOT: 88 of 1260 (7%)

Even though the type of TB or method of diagnosis was not specified in the selection criteria, the data extracted for all eight studies were for new, sputum smear-positive TB patients, treated with regimens for drug-sensitive TB. In some studies DOT was provided for only the intensive phase (first two months of treatment), and then only for the first dose each week during the continuation phase (all with thrice weekly treatment regimens [29-31]), or DOT was replaced by SAT during the continuation phase (daily treatment regimens [20,27]). In one study DOT was for daily dosing in the intensive phase only, followed by daily attendance at the clinic to collect drugs to be taken without supervision for the continuation phase (for both CB DOT and clinic DOT patients) [28]. For the other two studies DOT was provided for the entire course of treatment; one with a daily treatment regimen [13] and the other with a five day per week regimen [26].

Additional file 3 contains the extracted data (with results for the primary and secondary outcome) from all eight included studies.

### Study results

The results from the included studies are summarised in Table 1. Two studies showed increased treatment success with CB DOT compared to clinic DOT [28,30]. Five studies, including the RCT, showed no significant difference between the two DOT strategies for treatment success [13,20,27,29,31], and one study showed similar treatment success for newly diagnosed patients and improvement with CB DOT for patients that had previously undergone TB treatment [26]. In this last study, all retreatment patients received clinic DOT in the intensive phase of treatment (to receive streptomycin injections) and then their choice of CB DOT, clinic DOT or SAT during the 6-month continuation phase [26]. Based on our inclusion criteria, only the newly diagnosed patients, who were treated with different DOT strategies in the intensive phase, were included in this review.

#### Outcomes of meta-analyses

**Figure 2:**
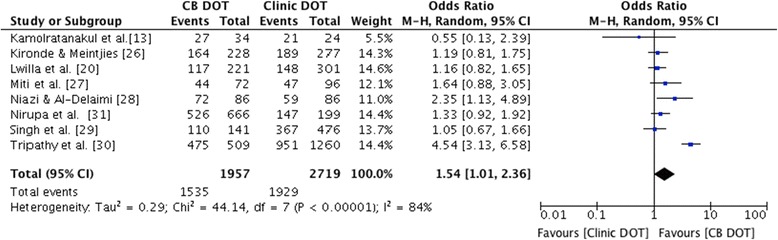
Forest plot of treatment success for CB DOT versus clinic DOT. The size of the symbols is proportional to the number of patients included in the meta-analysis.

The meta-analysis of all eight studies for treatment success showed that CB DOT was superior to clinic DOT (pooled OR of 1.54, 95% CI 1.01 – 2.36, p = 0.046, I^2^ 84% - Figure 2).

**Figure 3:**
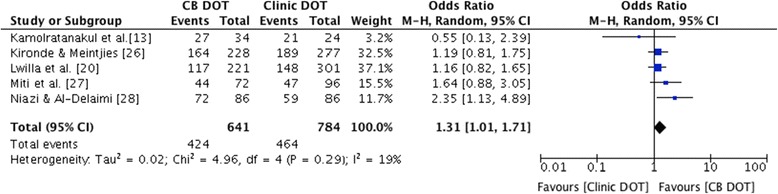
Forest plot of treatment success for CB DOT versus clinic DOT, prospective studies only. The size of the symbols is proportional to the number of patients included in the meta-analysis.

When only including prospective studies in the meta-analysis (Figure 3), there was markedly less heterogeneity among studies and again a significantly higher treatment success with CB DOT compared to clinic DOT (pooled OR 1.31, 95% CI 1.01-1.72, p = 0.045, I^2^ 19%).

**Figure 4:**
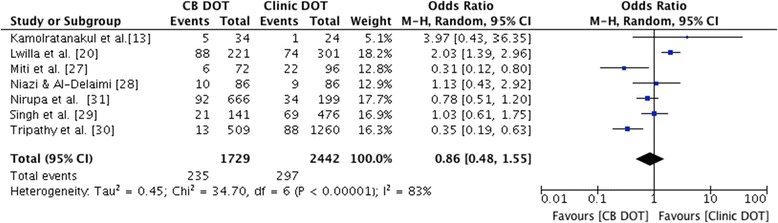
Forest plot of loss to follow-up for CB DOT versus clinic DOT. The size of the symbols is proportional to the number of patients included in the meta-analysis.

For the secondary outcome of loss to follow-up, data were available from seven studies. Comparing CB DOT with clinic DOT (Figure 4), the pooled OR was 0.86, 95% CI 0.48 to 1.55, and thus not significantly different (p = 0.62, with I^2^ heterogeneity 83%). This remained the case when only prospective studies were included for meta-analysis of loss to follow-up (OR 1.14, 95% CI 0.42-3.11, p = 0.8, I^2^ 79%) [13,20,27,28].

### Assessment of study quality and risk of bias

Based on the GRADE criteria [24], the quality of the included studies ranged from very low [13,26-29,31] to low [20,30]. The quality and the risk of bias assessments are provided in Additional file 4.

## Discussion

This systematic review and meta-analysis of eight studies that compared treatment outcomes of CB DOT with clinic DOT showed that CB DOT was associated with higher treatment success than clinic DOT. There was substantial inter-study heterogeneity associated with this pooled analysis. However, the benefit from CB DOT for treatment success compared to clinic DOT was also seen when including only prospective studies in the meta-analysis, with markedly less heterogeneity among studies. There was no significant difference between CB DOT and clinic DOT for the secondary outcome of loss to follow-up.

Strengths of our review are strict in- and exclusion criteria with clear definitions for CB DOT and clinic DOT, study selection and data extraction by independent reviewers and selection of the primary and secondary outcome prior to performing the review.

Our review also has several limitations. It is possible that we may have missed some studies despite a comprehensive literature search. The meta-analysis was limited by substantial inter-study heterogeneity. However, there was significantly less heterogeneity among prospective studies only, and a meta-analysis of this subgroup confirmed a higher treatment success rate with CB DOT compared to clinic DOT. Furthermore, the quality of the reviewed studies was generally low (see quality assessment in Additional file 4). As in all systematic reviews, reporting bias has to be considered. However, as there are less than ten studies in this meta-analysis, funnel plot asymmetry tests are not appropriate as “the power of the tests is too low to distinguish chance from real asymmetry” ([25], section 10.4.3.1). Following from these limitations, the result of the meta-analysis for treatment success needs to be interpreted with caution.

Studies in which CB DOT and clinic DOT programmes could not be directly compared (criterion 5), or where an allocation bias was evident (criterion 6), were excluded from this review (these are listed in Additional file 2). However, the included studies were still subject to risk of bias (see Additional file 4 for these assessments). Some points warrant further examination to ensure that the higher treatment success for CB DOT found in our meta-analysis was truly due to type of DOT supervision rather than due to systematic differences between the clinic DOT and CB DOT groups.

In the RCT from Tanzania, the largest treatment centre in the study was randomised to clinic DOT [20]. According to the study authors, this centre was likely to have had a higher proportion of patients with relatively more severe TB and thus with a lower likelihood of treatment success, compared to other study areas. This was a weakness of the cluster randomisation design and the authors note that their study “may have resulted in an overestimate of the benefits of CBDOT”. Other potential sources of bias in this RCT were unclear method of randomisation, inadequate (or unclear) allocation concealment and blinding of assessors, and a 31% loss to follow-up. This overall risk of bias means that this RCT constitutes relatively low quality evidence according to the GRADE criteria [24]. The study that allowed for supervisor self-selection for those patients randomly allocated to DOT (as opposed to SAT) was obviously prone to a substantial risk of selection bias [13]. Another important source of potential allocation bias was involvement of health workers in the decision who would receive CB DOT or clinic DOT [26,30,31]. None of these studies outlined any criteria to guide this allocation. While we cannot exclude that some of the potential selection biases discussed above impacted on overall treatment success for CB DOT and clinic DOT, none of the included studies had an apparent systematic selection bias for including patients in either the CB DOT or clinic DOT group, thus making it likely that the type of DOT was indeed the major cause of the difference in treatment outcomes.

While patient self-selection of DOT supervisor and/or consultation with a health worker adds potential study bias, patient-centred DOT allocation may be an important factor in treatment success. The authors of the study from Thailand, in which DOT led to higher cure and treatment completion rates than SAT, noted that: “Giving patients a variety of supervision options and focusing on their convenience may have contributed to the comparatively favourable results in this study” [13]. CB DOT removes the need to attend the clinic daily for TB treatment and this may help to address some of the barriers– which include time away from work–faced by patients receiving DOT [5]. Non-randomised studies where CB DOT and clinic DOT were assigned to patients from ‘unmatched’ geographical locations (e.g. rural for CB DOT versus urban for clinic DOT) were excluded from the review [12,49,50]. While this exclusion was made to increase the likelihood that differences in treatment outcomes were attributable to the DOT type rather than other factors, it may be that patients living greater distances from health facilities will benefit most from CB DOT being made available as a treatment option. Indeed, the results for CB DOT in terms of cure were at least equivalent to clinic DOT in each of these three studies [12,49,50]. While it may be beneficial in many cases, some risks, such as stigma, can still be associated with CB DOT. In the study from Zambia, many of the patients not previously registered with the ‘home care programme’ which delivered CB DOT, opted for clinic DOT instead (see risk of bias assessment in Additional file 4 for more details) [27]. This was due to the perception that community members would assume that they had HIV as well as TB if they saw community observers visiting them [27].

A limitation of the meta-analysis was that it did not allow to definitely establish the reason for increased treatment success among patients receiving CB DOT. Increased treatment success could be the consequence of lower rates of loss to follow-up (the secondary outcome for our study), lower rates of treatment failure or lower rates of death. While it is generally justified to assume that a significant proportion of treatment failures is caused by lack of adherence to TB treatment, undiagnosed drug-resistance of *M. tuberculosis* can substantially contribute to treatment failures in settings with relatively high proportions of drug-resistance in the community and lack of routine testing for drug-susceptibility [51]. However, it is unlikely that patients with epidemiological and medical risk factors for drug-resistant (DR) TB (such as retreatment cases, all data for meta-analyses were for newly diagnosed patients) were over-represented in the clinic DOT group, which would have affected treatment outcomes. This is again due to the exclusion of studies with apparent systematic selection bias for patient allocation to CB DOT or clinic DOT.

The forest plot for the secondary outcome - loss to follow up (Figure 4) - showed a wide spread of results. Brief discussion of the study showing higher loss to follow-up for CB DOT relative to clinic DOT in our meta-analysis is provided in Additional file 3 [20]. While numbers lost to follow-up can be seen as being patients who ‘defaulted’ on treatment, it is also possible that in some cases this outcome was a reflection of the quality of the research study as well as of outcomes of the DOT programme. The authors of the RCT report that the pragmatic nature of the trial “was largely the reason for a higher than-desirable number of missing outcomes” [20], reflecting the difficulties conducting this kind of research. In most of the included studies a specific definition for the loss to follow-up (or default) outcome was not provided [13,20,27,29,31]. Thus there is a risk that in some cases the patients assigned as being lost to follow-up were actually ‘not evaluated’ (e.g. ‘transferred out’) according to WHO treatment outcome definitions [21]. Even so, results for ‘transferred out’ were reported for all but one of the studies where the loss to follow-up definition was not specifically defined [31] meaning that this should have had a limited impact on the secondary outcome meta-analysis, and no impact on the treatment success meta-analysis result.

A Cochrane review assessed TB treatment success of DOT compared to SAT and if this was affected by the type of supervision provided in the DOT group [17]. The authors only identified one RCT that compared CB DOT and clinic DOT. This RCT- that we have also included in our meta-analysis- did not show any significant difference between CB DOT and clinic DOT in terms of cure [20].

A systematic review and meta-analysis published in 2009 examined the impact of different designs of CB DOT programmes on treatment outcomes [9]. Components of CB DOT programmes were categorised into patient, operational, and organisational characteristics. Studies in which the community-based supervisor was a health professional, family member, CHW or CV were included; though supervision needed to be at a place other than a health facility or ‘TB club’. It showed a possible (albeit plausibly due to chance) benefit from offering a financial reward to DOT supervisors (85.7% versus 77.6% for treatment completion, p = 0.15). The effectiveness in terms of treatment outcomes of CB DOT compared to clinic DOT was not examined in that review.

In the studies included in our systematic review, financial incentives to CHWs or CVs were not offered for providing DOT, or it was not stated whether these were provided or not (see Additional file 3 for further details about individual studies). However, using incentives as a motivator for DOT supervisors appears to be a common practice. This could be in the form of a regular salary, for example in the case of health extension workers in southern Ethiopia [35] or treatment supporters in South Africa [52]. Other forms of remuneration for treatment supervisors, sometimes involving patient co-payments, are also described in the literature [53].

Additional qualitative and quantitative aspects of community involvement in TB care have also been discussed in a review article and a WHO report [4,32]. These focused on the design of CB DOT programmes and their place in a wider policy context. Issues of accountability of CHWs or CVs, the need for training and variation in the quality of community-based supervision were raised by individual studies included in our review and these are important to consider during CB DOT programme design and monitoring.

While we acknowledge that the result of this systematic review has to be interpreted with caution, given the limitations outlined above, the result of higher treatment success is encouraging given the potential role of CB DOT as a means of treatment supervision, especially in resource-constrained settings.

CB DOT may also provide a means by which DR TB (multidrug-resistant (MDR)-TB or extensively drug-resistant (XDR) TB) can be supervised effectively, although all of the included studies in this review reported outcomes for patients treated with regimens for drug-sensitive TB. A recently published systematic review and meta-analysis – published prior to but indexed after our database search - has reported treatment outcomes from community-based DR TB programmes, though it did not compare the treatment success of CB DOT with conventional clinic DOT and none of the included studies were eligible for our review [54]. The review showed a pooled treatment success of 65% for 1,288 patients across ten studies with community-based interventions (including DOT by family members, neighbours, co-workers, local health care workers and former patients). This is comparable to outcomes seen in earlier meta-analyses of DR-TB treatment success [55-57].

## Conclusions

Our meta-analysis showed a benefit from CB DOT compared to clinic DOT for treatment success, but no overall difference between the two DOT strategies for loss to follow-up. This result in favour of CB DOT for treatment success was also seen when only analysing data from prospective studies. The lack of high quality RCTs limits the strength of this result. However, based on the review result, we suggest that CB DOT can be considered as an alternative to clinic DOT for the treatment of TB patients, delivering at least equivalent treatment success. Future studies should also assess the cost-effectiveness of CB DOT compared to clinic DOT, which is essential for health policy makers, especially in resource-constrained settings.

## Additional files

Additional file 1:
**Search of MEDLINE, EMBASE and PreMEDLINE.**
Additional file 2:
**Justification of studies excluded under criteria five and six.**
Additional file 3:
**Summary of individual studies.**
Additional file 4:
**Quality and risk of bias assessments.**

## Abbreviations

CB DOT: Community-based directly observed therapy
CHW: Community health worker
CI: Confidence interval
CV: Community volunteer
DOT: Directly observed therapy
DR TB: Drug-resistant tuberculosis
GRADE: Grading of Recommendations Assessment, Development and Evaluation
MDR TB: Multidrug-resistant tuberculosis
PRISMA: Preferred Reporting Items for Systematic Reviews and Meta-Analyses
OR: Odds ratio
RCT: Randomised controlled trial
RR: Risk ratio
SAT: Self-administered treatment
TB: Tuberculosis
WHO: World Health Organization
XDR TB: Extensively drug-resistant tuberculosis
