# Supplementary material for: Community-based directly observed therapy (DOT) versus clinic DOT for tuberculosis: a systematic review and meta-analysis of comparative effectiveness
Source: BMC Infect Dis. 2015 May 8;15:210. doi: 10.1186/s12879-015-0945-5 (PMC4436810; doi:10.1186/s12879-015-0945-5)
Supplement: Additional file 1: — Search of MEDLINE, EMBASE and PreMEDLINE. [file 12879_2015_945_MOESM1_ESM.docx]

**Additional file 1. Search of MEDLINE, EMBASE and PreMEDLINE.**

1. exp Community Networks/ or exp Community Health Services/ or exp Community Health Nursing/ or exp Health Services for the Aged/ or community care.mp. or exp Home Care Services/
2. exp Directly Observed Therapy/
3. DOT*.mp.
4. exp "Delivery of Health Care"/ or exp Community Health Services/ or exp Community Health Workers/ or exp Community-Based Participatory Research/
5. 1 or 2 or 3 or 4
6. exp Tuberculosis/dt, th [Drug Therapy, Therapy]
7. 5 and 6 🡪 output.
