# Supplementary material for: Community-based directly observed therapy (DOT) versus clinic DOT for tuberculosis: a systematic review and meta-analysis of comparative effectiveness
Source: BMC Infect Dis. 2015 May 8;15:210. doi: 10.1186/s12879-015-0945-5 (PMC4436810; doi:10.1186/s12879-015-0945-5)
Supplement: Additional file 2: — Justification of studies excluded under criteria five and six. [file 12879_2015_945_MOESM2_ESM.docx]

**Additional File 2: Justification of studies excluded under criteria five and six.**

| **Study [reference on main paper]** | **Rejection criterion** | **Justification** |
| --- | --- | --- |
| Balasubramanian VN, Oommen K, Samuel R: **DOT or not? Direct observation of anti-tuberculosis treatment and patient outcomes, Kerala State, India.** *Int J Tuberc Lung Dis* 2000, 4(5):409-13. [33] | 5 | This study aimed to identify the proportion of patients who did not receive DOT as allocated and the effect of this on treatment outcomes. While CB DOT and clinic DOT were both provided, the results are not separated to allow comparison of the two supervision types. |
| Cavalcante SC, Soares ECC, Pacheco AGF, Chaisson RE, Durovni B, DOTS Expansion Team: **Community DOT for tuberculosis in a Brazilian favela: comparison with a clinic model.** *Int J Tuberc Lung Dis* 2007, 11(5):544-9. [34] | 5 | In the clinic DOT areas: patients received DOT, bus tokens and food vouchers. In the CB DOT area: patients received DOT via CHWs, supportive social network, registering local organisations that offered social services (day care centres etc.). An NGO started a series of arts events to raise awareness of TB. Direct comparison of CB DOT and clinic DOT not reasonable. |
| Datiko DG, Lindtjorn B. **Health extension workers improve tuberculosis case detection and treatment success in southern Ethiopia: a community randomized trial.** *PLoS ONE* 2009, 4(5):e5443. [35] | 5 | In the intervention area (CB DOT) health extension workers (HEWs) provided with training in detecting TB cases, collect sputum and provide DOT during treatment (administered by HEWs at health posts). In the control areas, health extension workers were not trained and DOT was administered by health workers and health centres. Thus, intervention involved different aspects of TB control, detection, diagnosis and treatment and thus comparing this to clinic DOT arm is not reasonable. |
| Egwaga S, Mkopi A, Range N, Haag-Arbenz V, Baraka A, Grewal P, Cobelens F, Mshinda H, Lwilla F, van Leth F: **Patient-centred tuberculosis treatment delivery under programmatic conditions in Tanzania: a cohort study.** *BMC Med* 2009, 7:80. [36] | 5 | Home-based DOT primarily family member DOT. Results directly comparing CB DOT (via CHWs and CVs) and clinic DOT not provided. |
| Ferreira V, Brito C, Portela M, Escosteguy C, Lima S: **DOTS in primary care units in the city of Rio de Janeiro, Southeastern Brazil.** *Rev Saude Publica* 2011, 45(1):40-8. [37] | 5 | Nine units (of 32) offered DOT and patients could also choose SAT. One of the 9 units had a CHW in the team. No direct head-to-head comparison of CB DOT to clinic DOT provided. |

| Kangangi JK, Kibuga D, Muli J, Maher D, Billo N, N'Gang'a L, Ngugi E, Kimani V: **Decentralisation of tuberculosis treatment from the main hospitals to the peripheral health units and in the community within Machakos district, Kenya.** *Int J Tuberc Lung Dis* 2003, 7(9 Suppl 1):S5-13. [38] | 5 | Results showing a direct comparison between CB DOT and clinic DOT not provided. |
| --- | --- | --- |
| Mafigiri DK, McGrath JW, Whalen CC: **Task shifting for tuberculosis control: a qualitative study of community-based directly observed therapy in urban Uganda.** *Global Public Health* 2012, 7(3):270-84. [39] | 5 | Results showing a direct comparison between CB DOT and clinic DOT not provided (family members included as option for CB DOT, not separated from CHW or CV provided DOT). |
| Mahadev B, Kumar P, Sharada M: **How effective are shopkeepers as DOT providers? A study under RNTCP in Bangalore Mahanagar Palike, Karnataka**. *Indian J Tuberc* 2006, 53: 18-26. [40] | 5 | This study does compare CB DOT (delivered by shopkeeper) with clinic DOT. The number and proportion of patients for CB DOT, for clinic DOT and for new sputum smear-positive (NSP) TB overall are reported but not how many of each DOT supervision type had NSP TB is not clear. Clinic DOT was only offered to patients refusing CB DOT three times and thus if not rejected under criterion 5, this study would have been rejected under criterion 6c. |
| Mathema B, Pande SB, Jochem K, Houston RA, Smith I, Bam DS, McGowan JE: **Tuberculosis treatment in nepal: a rapid assessment of government centers using different types of patient supervision.** *Int J Tuberc Lung Dis* 2001, 5(10):912-9. [41] | 5 | Results showing a direct comparison between CB DOT and clinic DOT not provided (family members included as option for CB DOT, not separated from CHW or CV provided DOT). |
| Pungrassami P, Chongsuvivatwong V: **Are health personnel the best choice for directly observed treatment in southern Thailand? A comparison of treatment outcomes among different types of observers.** *Trans R Soc Trop Med Hyg* 2002, 96(6):695-9. [42] | 5 | This paper analysed the effect of the ‘practical observer’ of DOT (of health personnel, community member, family member, self-administration, mixed) and thus a head-to-head comparison of clinic DOT and CB DOT is not possible. |

| Pungrassami P, Johnsen SP, Chongsuvivatwong V, Olsen J: **Has directly observed treatment improved outcomes for patients with tuberculosis in southern Thailand?** *Trop Med Int Health* 2002, 7(3):271-9. [43] | 5 | This paper analysed the impact of whether DOT, as assigned, had occurred or not and a head-to-head comparison of clinic DOT and CB DOT is not reported. |
| --- | --- | --- |
| van den Boogaard J, Lyimo R, Irongo CF, Boeree MJ, Schaalma H, Aarnoutse RE, Kibiki GS: **Community vs. facility-based directly observed treatment for tuberculosis in Tanzania's Kilimanjaro Region.** *Int J Tuberc Lung Dis* 2009, 13(12):1524-9. [44] | 5 | Results showing a direct comparison between CB DOT and clinic DOT not provided (family members included as option for CB DOT, not separated from CHW or CV provided DOT). |
| Wandwalo E, Kapalata N, Egwaga S, Morkve O: **Effectiveness of community-based directly observed treatment for tuberculosis in an urban setting in Tanzania: a randomised controlled trial.** *Int J Tuberc Lung Dis* 2004, 8(10):1248-54. [14] | 5 | Results showing a direct comparison between CB DOT and clinic DOT not provided (family members included as option for CB DOT, not separated from CHW or CV provided DOT). |
| Yassin MA, Datiko DG, Tulloch O, Markos P, Aschalew M, Shargie EB, et al. **Innovative Community-Based Approaches Doubled Tuberculosis Case Notification and Improve Treatment Outcome in Southern Ethiopia.** *PLoS ONE* 2013, 8(5):e63174. [45] | 5 | Intervention package (CB DOT) included 1) capacity strengthening workshop; 2) advocacy, communication and social mobilisation component; 3) an active case finding component; 4) a communication and transport component and 5) a treatment component. “Hadiya zone, which did not receive the package, was used as a control zone to compare the outcomes”. Not reasonable to compare the two areas. |
| Zvavamwe Z, Ehlers VJ: **Experiences of a community-based tuberculosis treatment programme in Namibia: a comparative cohort study.** *Int J Nurs Stud* 2009, 46(3):302-9. [46] | 5 | Results showing a direct comparison between CB DOT and clinic DOT not provided (family members included as option for CB DOT, not separated from CHW or CV provided DOT). |
| Zwarenstein M, Schoeman JH, Vundule C, Lombard CJ, Tatley M: **A randomised controlled trial of lay health workers as direct observers for treatment of tuberculosis.** *Int J Tuberc Lung Dis* 2000, 4(6):550-4. [18] | 5 | Clinic DOT involved attendance to a health clinic 5 days per week for the first 8 weeks for new patients (12 weeks for re-treatment patients). “Patients on LHW [lay health worker] supervision took their drugs several times per week at their LHW’s home and under the LHW’s direct supervision”. Level of DOT is different and thus the two groups are incomparable. |

| Arora VK, Singla N, Gupta R: **Community mediated domicillary DOTS execution: a study from New Delhi**. *Indian J Tuberc* 2003, 50:143-150. [47] | 6a | **“**Patient selection [for CB DOT]: The study group was primarily constituted by those TB patients attending out-patient department of Institute, who were offered and even motivated to take DOTS but on their own gave reasons for the non-acceptance. These comprised of either inconvenient centre timings (menacing with loss of hours of job, study or house-hold work), unavailability of nearby DOTS centres (resulting in unbearable travel costs), patient disabilities (making regular centre-visits difficult) or social stigma.” Comparison between CB DOT and clinic DOT (institute’s DOTS centre) not reasonable. |
| --- | --- | --- |
| Bhagyalaxmi A, Jain S, Kadri AM: **Effectiveness of Different Models of DOTS Providers under RNTCP in Ahmedabad City, Gujarat.** *Indian J Community Med* 2010, 35(4):495-7. [48] | 6c | From the methods section: “Maximum effort was made to include only category I patients, however, in some of the centers new cases were not supervised by non-TBHVs [CB DOT], in such cases, categories II and III were also included in the study.” For TBHV (clinic DOT) category I, N = 81 (61.83%), category II, N = 7 (31.8%) & category III, N = 17 (36.17%). For non-TBHV (CB DOT) category I, N = 50 (38.17%), category II, N = 15 (22.72%) and category III, N = 30 (63.83%). An allocation bias based on type of TB is apparent, therefore excluded. |
| Khieu K, Ito K, Hamajima N: **Experience in tuberculosis treatment through directly observed therapy short course in health centres and communities in Cambodia.** *Public Health* 2007, 121(9):696-9. [49] | 6b | CB DOT provided only in [from methods] “communities located in rural areas far from the health centres in the same two districts”. [from discussion] “ The criteria for the people with TB who could receive the treatment in their own villages were those who lived far from the health centre (more than 3 km) or those who could not afford to travel to the health centre every day.”  As patients in the CB DOT group lived 3 to 20km from health centres, differences in access to healthcare relative to the clinic DOT group may have accounted for any differences in treatment outcomes. The two groups were similar based on sex, age and type of TB (sputum-positive, negative or extra-pulmonary), but measures related to the socioeconomic status of each group were not provided. |
| Mahyiuob Al-Honahi HY, Ohkado A, Masui T, Ali-Hussein IA, Saeed Al-Absi AN: **A trial to mobilize NGO health volunteers to improve tuberculosis patient care in Sana'a City, Yemen.** *Kekkaku* 2010, 85(3):159-62. [50] | 6b | [From methods section] “1) TB patient who lives nearby (roughly within 20 minutes walking distance) a hospital/health centre is encouraged to come to the nearest health facility every day during the intensive phase of treatment; 2) TB patient who lives far from the health facilities has an NGO’s health volunteer as a DOTS partner.” Again differences in healthcare access may have affected treatment outcomes in addition to type of DOT provided, though no socioeconomic data is provided to assess differences. |
| Walley JD, Khan MA, Newell JN, Khan MH: **Effectiveness of the direct observation component of DOTS for tuberculosis: a randomised controlled trial in Pakistan.** *Lancet* 2001, 357(9257):664-9. [12] | 6b | Clinic DOT eligibility criteria was “that the return journey from the patient’s home to the health facility was a distance of less than 2km, a duration of less than 2 hours and a cost of less than 10 rupees; and for unmarried women, an accompanying relative was to be available”. If this criteria was not met, CB DOT via CHW allocated. The CB DOT and clinic DOT groups were from different geographical areas and, as for the two studies above, may face barriers to health care access. Odds ratios of cure and treatment success were reported adjusted for age and sex, however these are reported for health workers (including clinic staff and community health workers) overall and not separately for CB DOT and clinic DOT. |
