# Supplementary material for: Community-based directly observed therapy (DOT) versus clinic DOT for tuberculosis: a systematic review and meta-analysis of comparative effectiveness
Source: BMC Infect Dis. 2015 May 8;15:210. doi: 10.1186/s12879-015-0945-5 (PMC4436810; doi:10.1186/s12879-015-0945-5)
Supplement: Additional file 3: — Summary of individual studies. [file 12879_2015_945_MOESM3_ESM.docx]

**Additional file 3. Summary of individual studies.**

| **Authors and year of publication [study ref.]** | **Kamolratanakul *et al.* 1999 [13]** |
| --- | --- |
| Study Objective | To compare DOT with self-administered treatment. |
| Setting (rural, urban or mixed) | Thailand (mixed). |
| RCT or non-randomised (observational/interventional) | RCT- for DOT arm supervisor self-selected. |
| New, sputum-smear positive (NSP) results reported (yes/no) | Yes |
| Study design and description | 15 study sites (across Thailand): 8 district hospitals, 3 provincial hospitals, 4 TB referral centres. Patients randomised to intervention or control group after diagnosis and cases meetings, exclusion criteria were excluded. Daily treatment regimen **Intervention group:** DOT throughout 6-month treatment by patient supervisor - patient could choose between health centre staff, community volunteers (e.g. village health volunteers or other community leaders) or family members. Community volunteer or family member (if chosen) received treatment card, visited by health centre staff fortnightly (2 months), then monthly (monitoring from treatment card, pill counts, urine tests - visual for rifampicin red). **Control group (prevailing method):** monthly drug supply, no treatment supervision and monthly clinic visits. Primary outcome cure, secondary treatment completion and sputum conversion (intention-to-treat analysis). |
| Incentives to CHW/CV (yes/no) | None mentioned. |
| Study period | August 1996 - October 1997. |
| Number of participants | 837 |
| Inclusion Criteria | New, sputum-smear positive pulmonary TB. |
| Exclusion Criteria | Age < 15 years, retreatment patients, known allergies to standard drug regimen (2 months daily rifampicin (R), isoniazid (H), pyrazinamide (Z) and ethambutol (E), 4 months daily RH), pregnancy, hepatic or renal failure. |
| Ethics approval (yes/no/not stated) | Patients not informed about the study and the existence of alternative treatment options, ethical approval not mentioned. |
| Control group | SAT |
| Outcomes | Primary outcome: cure, defined as percentage of patients who completed 6 months of treatment and had 2 negative sputum examinations during treatment.  Secondary outcomes: treatment completion (defined as percentage who completed 6 months of treatment but did not have 2 negative sputum examinations during treatment) and sputum conversion (defined as percentage of patients who were smear-negative for acid-fast bacilli at the end of the third month of treatment). Other outcomes were measures proportion of patients defaulting from treatment, died or transferred to different treatment locations (definitions for these not provided). |
| Results | One eligible patient did not receive DOT as allocated, 836 patients followed up, 414 in intervention and 422 in control group. No significant socio-demographic difference between groups. In intervention group 315 (76%) were cured, 347 (84%) completed treatment and 27 (7%) defaulted. For control group, cure was achieved for 283 (67%) patients, treatment completion for 320 (76%) and default 55 (13%). Significant difference for all (and transfer out), but not for death, treatment failure or sputum conversion (at the end of month 3). Greater difference between groups at hospitals (district and provincial) relative to TB referral centres. In intervention group, supervision type was known for 410 patients (99%). Health worker supervision (N=24) yielded 19 (79%) patients cured; 21 (88%) completing treatment and 1 (4%) defaulting. Community member (N=34) supervision led to 25 (74%) cures, 27 (79%) treatment completions and 5 (15%) defaults. Family members (N=352) yielded 270 (77%) cures, 297 (84%) completions and 21 (6%) defaults. Differences between supervision groups were non-significant. |
| Limitations | Pre-study results in hospitals were: cure 48%, completion 64%, default 23%. In TB referral centres: cure 67%, completion 74% and default 12%. Apparent Hawthorne effect for both intervention and control arm during study. |
| Main conclusion(s) | DOT was more effective than SAT, but less so a TB referral centres than hospitals. DOT appears dependent on a de-centralized approach. Non-significant difference in treatment outcomes between three types of treatment supervisors, though family seems the preferred option for most patients. |
| Funding sources | INCLEN |

| **Authors and year of publication [study ref.]** | **Kironde and Meintjies. 2002 [26]** |
| --- | --- |
| Study Objective | To determine the effect of the choice of treatment delivery option on treatment outcomes. |
| Setting (rural, urban or mixed) | South Africa (mixed). |
| RCT or non-randomised (observational/interventional) | Prospective cohort study. |
| NSP results reported (yes/no) | Yes |
| Study design and description | 45 primary health clinics used for the study. Patient given option of clinic or community DOT. If neither is feasible (if long distance from clinic or no treatment supporter available), then SAT is offered. Five day a week treatment regimen. **Clinic DOT:** patients attend clinic 5 days per week. **Community DOT:** assigned to lay health worker (LHW), patient usually goes to supporter’s house 5 days per week. LHWs given a secure box for drugs, record cards and basic training is provided. Max 4 patients per LHW. **SAT:** patients given monthly drug supply and record card. **Retreatment patients** receive clinic DOT for two months (due to streptomycin injections) and choice thereafter. Data collection through TB registers, pre-coded survey (in Afrikaans) at commencement and follow-up interviews during treatment. |
| Incentives to CHW/CV (yes/no) | None mentioned. |
| Study period | October 1999-October 2000 |
| Number of participants | 769 |
| Inclusion Criteria | Patients > 15 years with pulmonary TB confirmed by sputum smear microscopy, new TB patients starting treatment from October 1999-Feb 2000 and retreatment patients between November 1999 and October 2000. |
| Exclusion Criteria | Not stated. |
| Ethics approval (yes/no/not stated) | Yes and patient consent obtained. |
| Control group | SAT |
| Outcomes | Treatment outcome: successful (**cured** – complete treatment and had a negative sputum-smear at the end of treatment and on at least one previous occasion, or **completed** – as for cure, with a good clinical response, but with no sputum investigations at the end of treatment) or unsuccessful (**default** – patients failing to take their treatment for 2 consecutive months or longer, **failure** – patients completing a course of treatment but still being sputum smear-positive at its completion, **died** – from any cause during treatment, **transferred** – patients moved to another health facility outside of the study area). |
| Results | 719 patients included in analysis; of these 598 were new and 121 were retreatment (50 transferred out and were not included in analysis). Treatment success for LHW DOT was 72% (164 of 228) for new patients and 76% (19 of 25) for retreatment. For clinic DOT treatment success was 68% (189 of 277) for new and 65% (41 of 64) for retreatment. For SAT patients, treatment success was 71% (66 of 93) for new and 16% (5 of 32) for retreatment. Differences between groups were non-significant for new patients (p=0.671) and significant for retreatment (p<0.001). New patients had twice the chance of treatment success relative to retreatment. For new patients, rural residence, secondary level education and knowledge about treatment duration were associated with higher likelihood of treatment outcome, while living in a shack decreased success likelihood. In multiple logistic regression model, supervision type for new patients was still not significant (p=0.875). For retreatment patients, no difference in sex or age relative to new patients. Supervised retreatment patients more likely to be successful than SAT (odds ratio (OR) 14.2, 95% CI 4.18-53.14). Reporting to government health unit when ill also associated with better outcome. In multiple logistic regression model, choice of supervision type significantly associated with outcome. First attendance at government health unit no longer significant. No significant association between receiving a disability grant and/or side effects (same as in univariate analysis) and treatment success. Retreatment patients sub-analysis performed: RC - relapse patients previously declared cured had treatment success almost as high as new patients (78.9%). Rural residence associated with higher likelihood of success. Secondary education and knowledge of how long treatment should go for were also independently associated with better outcome. 19% of those interviewed did not know the treatment duration. Default rates of 18.7% reported but not broken down in terms of allocation type. |

| Limitations | Almost a quarter of patients (194 of 769) defaulted or transferred elsewhere, association of HIV not explored though authors suggest that this could be a contributor to high proportion defaulting/transferring out. |
| --- | --- |
| Main conclusion(s) | DOTS should be prioritised for retreatment patients (especially RI - retreatment after default and RF - retreatment after failure) and a menu of options should be available to patients. |
| Funding sources | Sir Halley Stewart Trust (UK) and the Department for International Development—Southern Africa (DFID UK) |

| **Authors and year of publication [study ref.]** | **Lwilla *et al*. 2003 [20]** |
| --- | --- |
| Study Objective | To assess whether for patients with new, sputum smear-positive pulmonary TB, smear conversion at 2 months and treatment outcomes were similar using community-based DOT (CBDOT) and institutional DOT (IBDOT). |
| Setting (rural, urban or mixed) | Tanzania (rural). |
| RCT or non-randomised (observational/interventional) | Open cluster RCT. |
| NSP results reported (yes/no) | Yes |
| Study design and description | 18 TB units paired based on type of health facility & number of TB patients registered in previous year. One unit within each pair randomly assigned to CB-DOT or IB-DOT. DOT during intensive phase only (daily treatment regimen). **For CB-DOT:** a community member from the same village observed the patient during the 1st 2 months. Observer selected by village leaders and trained by District TB and Leprosy coordinator (DTLC), observers visited fortnightly by health worker and monthly by DTLC. **For control arm:** patient had to visit health facility daily for first 2 months. CBDOT visit health clinic once per month, IBDOT likewise (although every day during intensive phase). SAT during continuation phase. At 2, 5, 7 months sputum taken for smear microscopy. Intention-to-treat analysis, sensitivity analyses performed to assess potential impact of missing responses. Sex and age data also collected. |
| Incentives to CHW/CV (yes/no) | No |
| Study period | Jan 1999 and June 2000. |
| Number of participants | 522 |
| Inclusion Criteria | NSP TB patients, treated with ambulatory short-course therapy. |
| Exclusion Criteria | Not stated |
| Ethics approval (yes/no/not stated) | Yes and verbal consent obtained. |
| Control group | IBDOT patients. |
| Outcomes | Primary outcome: sputum conversion at 2 months.  Secondary outcome: cure rate at 7 months (for those sputum negative at 2 and remaining so at 5 and/or 7 months after the start of treatment, though note treatment is of 8 months duration). Other outcomes reported were transfer out, loss to follow-up, died and treatment failure (definitions not provided). |
| Results ^#^ | Groups at baseline: similar demographic profile. 1, 619 participants registered during study period: 522 with NSP TB were prescribed ambulatory treatment. IBDOT = 301, CBDOT =221. Completeness of follow up: 455 (of 522) at 2 months and 311 (of 473) at 7 months. Loss to follow up IBDOT: 33 (of 301), CBDOT: 24 (of 221) at 2 months. Significantly more losses in Mlimba-Kidodi and Idete-Zignalie areas. At 7 months: loss to follow up IBDOT 74 (of 259), CB DOT 88 (of 214), a significant difference. Heterogeneity between groups persisted. Fewer CBDOT patients died (p < 0.001). Conversion rates similar at 2 months. Not affected by sex or impacted by incomplete follow up. 7 months: cured patients similar (148 of 301 for IBDOT versus 117 of 221 for CBDOT, p =0.57). Sex and sensitivity analysis did not have great impact. |
| Limitations | Largest area with TB treatment hospital was randomised to IBDOT: sicker patients therefore risk of selection bias. Significant difference in mortality rates between arms may be because of this also - though this result remained when this hospital's patient data was removed from analysis. Heterogeneity; some community observers better than others. |
| Main conclusion(s) | Similar results for CBDOT versus IBDOT. CBDOT viable option given over-stretched health facilities. Non-inferior status of CBDOT -useful in Tanzania, especially when patients live >10km from health facility. Choice for patients is important, not a replacement for IBDOT, just another option. |
| Funding sources | The Tanzanian Health Users Research Fund, the Spanish Agency for International Co-operation (AECI), Fons Catalan and La Caixa |

# - Due to cluster (rather than individual) randomization for this RCT, the authors of this study performed a pooled estimate of eight paired clusters (of nine pairs, one was unpaired owing to one area not having a TB patient) using the Mantel-Haenszel method. For our meta-analysis, patient-level data has been taken as total cure at 7 months for CB DOT and clinic DOT (for all the study areas combined) and the odds ratio (OR) for this trial calculated based on these data. Thus, the ORs reported in their paper (OR 1.58, 95% CI 0.32-7.88) and in our meta-analysis (OR 1.16, 95% CI 0.82-1.65, both in favour of CB DOT) are different to one another due to unavailability of raw data to attempt to account for the impact of different treatment areas in our meta-analysis. For loss to follow-up, numbers for CB DOT and clinic DOT at 7 months considered as proportion of the original sample size of 522 in our meta-analysis (clinic DOT 301 and CB DOT 221), rather than 473 (259 clinic DOT and 214 CB DOT) reported in the paper, owing to insufficient data to rationalise the 473 patient number provided. The OR for loss to follow-up is 1.52, 95% CI 0.73-1.14 (p = 0.26) in the results table of the study (presumably of the total 522 patients, as an OR of 1.92, 95% CI 1.29-2.8 is reported for the reduced sample of 473 patients); our meta-analysis using individual patient data yielded an OR of 2.03, 95% CI 1.39-2.96, again all with CB DOT considered the intervention and clinic DOT the control.

| **Authors and year of publication [study ref.]** | **Miti *et al.* 2003 [27]** |
| --- | --- |
| Study Objective | To evaluate implementation of DOTS strategy as part of existing HIV/AIDS Health Care Program by comparing treatment outcomes between an intervention and control population. |
| Setting (rural, urban or mixed) | Zambia (urban). |
| RCT or non-randomised (observational/interventional) | Non-randomised trial. |
| NSP results reported (yes/no) | Yes |
| Study design and description | **In Twapia (control):** receive clinic DOT during intensive phase, during continuation phase dispensed weekly, fortnightly or monthly. In **Chipulukusu (intervention):** patients registered with home care programme received home-DOT from 'personal volunteer', during at least intensive phase. During continuation phase, same protocol as in control population. Sputum collection at 2, 5 and 8 months. Sputum samples collected from both townships daily and results returned the following day. Health care professionals & volunteers asked if they wanted to participate. 15 (one-third) volunteered and received a 3-day training course on TB management. Nurses from Twapia also underwent a short training course. Age, sex data also collected. Daily treatment regimen. |
| Incentives to CHW/CV (yes/no) | None mentioned |
| Study period | 1 February 1998 until September 30 1999. |
| Number of participants | 168 |
| Inclusion Criteria | All NSP TB patients, ≥15 years, registered during study period. |
| Exclusion Criteria | All others. |
| Ethics approval (yes/no/not stated) | Not stated. |
| Control group | Patients receiving clinic DOT (intensive phase) then SAT (for both arms, continuation phase). |
| Outcomes | Treatment outcome - cured, treatment completed, died, failure, treatment interrupted (defaulted) or transfer were reported outcomes; the definitions for these outcomes are not defined specifically in the paper. Aim for success of 80% for new, adult sputum-smear positive PTB and default rates of < 10%. |
| Results | Twapia (control), N = 96 at commencement, Chipulukusu (intervention), N = 72. Treatment success: control 49% (47) intervention 61% (44) (no significant difference, chi-squared 2.43, p <0.118), deaths (control 19%, N = 18, intervention 22%, N = 16) not significant (p-value not given). Most deaths during intensive phase. Cure rate: control 21% (20), intervention 54% (39) significant difference - p<0.001). Treatment interruption: control 23% (22), intervention 8% (6) - p <0.012. Transfer out similar between populations (8.3% intervention, control 9.4%). More sputum submission in intervention population. Failed to achieve success rate of 80% (largely due to high death rate for both populations). |
| Limitations | High death rate due to high HIV prevalence. |
| Main conclusion(s) | Volunteers satisfied with their work, made up for the extra work required. High death rates the major issue, possible to expand this program to other areas. No significant effect on treatment completion but significant effect on treatment interruption (lower in intervention group) and cure. |
| Funding sources | None mentioned, though attendance at WHO workshop for the ‘Community tuberculosis care in Africa’ programme during planning phase, unclear whether direct funding was provided for the study from the WHO or from the organisation’s other funding bodies. |

| **Authors and year of publication [study ref.]** | **Niazi and Al-Delaimi, 2003 [28]** |
| --- | --- |
| Study Objective | Assess the effect of home visits and support by a local NGO on outcomes of DOTS therapy: sputum smear conversion, cure and default rates and patient compliance with treatment. |
| Setting (rural, urban or mixed) | Iraq (urban). |
| RCT or non-randomised (observational/interventional) | Non-randomised trial (sequential allocation to one treatment arm or the other). |
| NSP results reported (yes/no) | Yes |
| Study design and description | Patients enrolled during the study period were alternately allocated to the intervention or control group. 2 months of DOT, 4 months continuation unsupervised (for both groups). **Intervention group** received daily home visits from trained members of the Iraqi Women's Federation (IWF) during 2-month intensive phase. During continuation phase they were expected to attend primary health centre daily to collect drugs and take them without supervision. 20 women from IWF trained for 2 days: responsibility for DOT and for monthly follow up during continuation phase. These members also collected some socio-demographic data for the study. Treatment outcome and compliance monitored by study coordinator (for both groups). **Control:** patients attend clinic daily for 6 months (DOT for 2 months, then 4 months unsupervised). |
| Incentives to CHW/CV (yes/no) | None mentioned. |
| Study period | February - December 2001 |
| Number of participants | 172 |
| Inclusion Criteria | TB patients newly diagnosed by the TB Institute, Baghdad, using the WHO criteria of 3 consecutive positive smears. |
| Exclusion Criteria | Not stated. |
| Ethics approval (yes/no/not stated) | Not stated. |
| Control group | Clinic DOT. |
| Outcomes | Sputum conversion rate: samples at end of 1, 2, 3 and 5 months.  Cure rate: percentage of patients who were sputum smear-negative in the last month of treatment and on at least one other occasion.  Treatment failure rate: percentage of patients who remain sputum smear-positive at 5 months or later during treatment.  Mortality rate  Defaulter rate: percentage of patients who did not collect their medication for 2 consecutive months or more.  Treatment outcome: cure, treatment failure, died and default.  Non-compliance: measured as numbers of days where doses were missed. |
| Results | N = 86 for control and for intervention. No significant difference in age, sex, type of family, occupation. More people in the intervention group owned their houses (79.1% vs 57%) and the crowding index was higher for the control (4.36 vs intervention with 3.73) both significant. Intervention versus control results: Sputum conversion: 1 month (86.1% vs 64% p =0.0015), 2 month (90.7% vs 77.9%, p = 0.036), 3 month (94.2% vs 79.1%, p = 0.0071), 5 month (97.7% vs 80.2%, p = 0.0006), Treatment outcome: cured 72 (83.7%) vs 59 (68.6%), p = 0.02), failed (3.5% vs 20%, p = 0.0009), defaulted 10 (11.6%) vs 9 (10.5%), p>0.05, died (1.2% vs 1.2%, p>0.05). Non-compliant with treatment (0% was 86.1%) - defaulters not considered as non-compliant. Significant inverse relation between number of missed doses and cure rate, cut off point ≥ 8 days. Major flaw in contact tracing, > 60% had index case. No socio-demographic factors associated with non-compliance. |
| Limitations | Did not measure other factors that may impact on compliance such as time to reach the health facility, waiting time, satisfaction with patient care received. |
| Main conclusion(s) | Treatment at home from trained NGO members improved cure, sputum smear conversion and compliance rates. |
| Funding sources | WHO Eastern Mediterranean Region (EMRO), Division of Communicable Diseases (DCD) and the WHO Special Programme for Research and Training in Tropical Diseases (TDR): the EMRO/DCD/TDR Small Grants Scheme for Operational Research in Tropical and Communicable Diseases |

| **Authors and year of publication [study ref.]** | **Nirupa *et al.* 2005 [31]** |
| --- | --- |
| Study Objective | To assess the treatment outcomes and problems encountered for patients with different types of DOT supervision. |
| Setting (rural, urban or mixed) | India (rural). |
| RCT or non-randomised (observational/interventional) | Retrospective cohort study. |
| NSP results reported (yes/no) | Yes |
| Study design and description | Four types of supervisors used (assigned by medical officers) 1) health workers from the government primary health institutions (PHIs) (pharmacist, lab technicians), 2) outreach workers (auxiliary nurse midwives, village health nurses and health inspectors, 3) Anganwadi health workers providing non-formal education to pre-school children in the village under the integrated child development services scheme 4) community volunteers such as religious leaders, school teachers, shopkeepers residing in patient's village or nearby village. Some patients received SAT (though not encouraged). Treatment cards used to obtain information. Type of provider verified over the phone by paramedical workers. DOT for each dose during intensive phase and then at least first weekly dose during continuation phase (thrice weekly regimen). |
| Incentives to CHW/CV (yes/no) | None mentioned. |
| Study period | May 1999 to June 2002 |
| Number of participants | 3,019 |
| Inclusion Criteria | For results: NSP pulmonary TB patients. |
| Exclusion Criteria | Not stated. |
| Ethics approval (yes/no/not stated) | Not stated. |
| Control group | SAT |
| Outcomes | Treatment outcome: treatment success (included patients who were cured or those who completed treatment), default, died, failure. The definitions for these outcomes are not defined specifically in the paper.  Also recorded type of DOT provider whether there were problems with drugs during treatment. |
| Results | 2,661 of 3,019 registered during study period (88%) could be contacted: 543 (20%) PHI DOT, 561 (21%) for government outreach workers, 1118 (42%) from Anganwadi workers, 377 (14%) from community volunteers, 62 (2%) SAT. Of total 3019, Median age 43, 2,237 (74%) male, 1,820 (68%) were employed. Patients assigned to different providers had similar socio-demographic characteristics (univariate analysis). Treatment outcome for 1,131 NSP patients provided with category 1 treatment. Of these, 199 (18%) PHI DOT, 238 (21%) from outreach workers, 496 (44%) from Anganwadi workers, 170 (15%) from community volunteers, 28 (2%) SAT. 864 (76%) male and 782 (69%) were employed. Treatment success, 147 (74%) PHI, 193 (81%) outreach workers, 397 (80%) anganwadi workers, community volunteers 129 (76%). Not statistically significant difference. SAT 75% treatment success. Community volunteer DOT default of 18%, PHI DOT 17%. Patients with SAT significantly more likely to have treatment failure or die. |
| Limitations | Info gaps (couldn't contact 12% of patients by phone). Patients that couldn’t be contacted more likely to have poorer outcomes than those that could be. Therefore success rates might be higher than actually the case. Potential that those receiving DOT were not actually being observed while taking their anti-TB drugs. Potential effect of other confounders not explored in the paper. |
| Main conclusion(s) | Possibility for involvement of Anganwadi workers/community volunteers (in addition to government staff) and reinforces importance of DOT (vs SAT) in this part of India. |
| Funding sources | United States Agency for International Development provided through the World Health Organization, SEARO, New Delhi |

| **Authors and year of publication [study ref.]** | **Singh *et al.* 2004 [29]** |
| --- | --- |
| Study Objective | To compare treatment success of patients followed by community volunteers versus government health workers. |
| Setting (rural, urban or mixed) | India (urban). |
| RCT or non-randomised (observational/interventional) | Retrospective cohort study. |
| NSP results reported (yes/no) | Yes |
| Study design and description | Community volunteer considered as non-government health workers who did not practice modern medicine (including shop owner, teacher etc.). Received two days training at district TB centre, workplaces used as 'DOT' centres. Patients had option of community volunteer supervision if one was located nearby. Observation thrice weekly during intensive phase (each dose) and then at least once weekly during continuation phase. If deviation treatment supervisor visited every 2-3 days and supervisor not assigned more patients, default tracing also by supervisor (most community volunteers in full-time employment), doctor visit once monthly and phone contact in between. Outcomes from TB treatment registers (thrice weekly treatment regimen). |
| Incentives to CHW/CV (yes/no) | No |
| Study period | April 2000 - Dec 2001. |
| Number of participants | 617 |
| Inclusion Criteria | NSP pulmonary TB. |
| Exclusion Criteria | All other types of TB. |
| Ethics approval (yes/no/not stated) | No |
| Control group | Government health worker provided DOT. |
| Outcomes | Treatment success = cure (2 negative sputum smears, one of these at the completion of treatment) + treatment completed (negative smears at the end of the initial phase but none were examined at the end of treatment). Other outcomes reported were died, failed, default, transferred (specific definition for these not provided – outcomes reported in TB registers). |
| Results | Total N = 617, community volunteer supervision, N = 141 and government health worker, N=476. Median 10 patients per volunteer (though range 2-53 and 5 volunteers supervised > 35 patients). Proportion of patients with volunteer supervision was 13% in 2000 and 25% by 2002. Treatment success was 110 (78%) for community volunteers and 367 (77%) for government health workers, p =0.9. Default was 15% for each, p = 0.62. |
| Limitations | Potential bias due to urban setting, selection of patients, selection of community volunteers, or motivation of staff (cited as an example in other districts). |
| Main conclusion(s) | Similar success rates with both arms. Since 2002: 1 TB Health visitor/150,000 urban residents (to help coordinate community volunteers). Supervisor follow up of defaulters may be more difficult in rural areas. Further study to see if success rates maintained if volunteer is responsible for follow up as well as treatment. |
| Funding sources | None mentioned |

| **Authors and year of publication [study ref.]** | **Tripathy *et al.* 2013 [30]** |
| --- | --- |
| Study Objective | To find out 1) whether community DOT (CDOT) is associated with equally favourable treatment outcomes compared to clinic DOT and 2) the profile for CDOT providers in an urban setting in Bangalore. |
| Setting (rural, urban or mixed) | India (urban). |
| RCT or non-randomised (observational/interventional) | Retrospective cohort study. |
| NSP results reported (yes/no) | Yes |
| Study design and description | DOT during intensive phase and at least the first dose during the continuation phase. Thrice weekly treatment regimen throughout. Patient chooses type of DOT provider. All providers, including community volunteers were trained. Treatment details, type of DOT provider and treatment outcome were recorded in a standard routine treatment card to each TB patient. Community DOT provider is a person who volunteered to administer DOT as per programme guidelines and who belonged to the community where the patient resided but was not a government health worker nor a member of the family. An institutional DOT provider is defined as a government health worker in the institution administering DOT. Demographic data including: age, sex and HIV status was collected. Sputum smear grade, sputum conversion, treatment duration and outcome collected. Data obtained from TB register included outcome of treatment, sputum smear grade and conversion. DOT provider obtained from clinic responsible for TB treatment. |
| Incentives to CHW/CV (yes/no) | None mentioned. |
| Study period | October 1, 2010-September 30, 2011 |
| Number of participants | 2099 |
| Inclusion Criteria | NSP pulmonary TB. |
| Exclusion Criteria | Not stated. |
| Ethics approval (yes/no/not stated) | Yes |
| Control group | Institutional DOT (IDOT). |
| Outcomes | Sputum smear conversion and treatment outcome (reported as cured, treatment completed – both‘favourable’, and failure, defaulted, died, transferred out – ‘unfavourable’. Definitions “used routinely in the RNTCP [revised national TB control programme]”. |
| Results | Treatment cards of 1,864 available for evaluation. 604 (32%) CDOT, 1260 (68%) IDOT. Favourable outcome CDOT: 564 (93%), IDOT 951 (75%), Unfavourable outcome: CDOT 40, (7%), IDOT 309 (25%). No significant difference between age group, sex or initial sputum grade. Of 57 HIV patients 23% underwent CDOT. Significant difference between CDOT and IDOT in outcomes between default (2.6% versus 7%) and failure (0.8% versus 4.1%). Higher in IDOT for both. No significant difference between CDOT providers (CVs, N = 430, physicians N = 95, alternative medicine providers, N = 12, shopkeepers, N = 16, teachers, N = 2 and 'others' (community members or colleagues), N = 49. In IDOT, TB health visitors, staff nurses, auxiliary nurse midwives, others (including lab technicians and attendants). Detailed results for each provider type given. No significant difference between TB health visitors and all other DOT providers. |
| Limitations | Relies on accuracy of records, “which are not always free of errors”. Other confounding factors might affect treatment outcomes. If patients change DOT provider during treatment, this was not captured in the record. |
| Main conclusion(s) | CDOT associated with more favourable outcomes relative to IDOT. |
| Funding sources | USAID |
