# Supplementary material for: Community-based directly observed therapy (DOT) versus clinic DOT for tuberculosis: a systematic review and meta-analysis of comparative effectiveness
Source: BMC Infect Dis. 2015 May 8;15:210. doi: 10.1186/s12879-015-0945-5 (PMC4436810; doi:10.1186/s12879-015-0945-5)
Supplement: Additional file 4: — Quality and risk of bias assessments. [file 12879_2015_945_MOESM4_ESM.docx]

**Additional File 3. Risk of bias and quality assessments.**

**Risk of bias assessment (RCT)**

| **Trial [reference]** | **Randomization type** | **Allocation sequence generation** | **Allocation concealment** | **Blind (assessors)** | **Completeness of follow up** |
| --- | --- | --- | --- | --- | --- |
| Lwilla *et al.* 2003 [20] | Cluster – 9 matched pairs, random assignment within pairs. | Unclear – method of random assignment is not provided. | Unclear – though does say unmasked, so expect to be inadequate. | Inadequate | Inadequate – 31% loss to follow-up overall. Not clear which are ‘defaults’ and which are not kept track of. |

**Risk of bias assessment (non-randomised studies)**

| **Study [ref.]** | **Potential allocation bias** | **Potential reporting bias** | **Additional comments** |
| --- | --- | --- | --- |
| Kamolratanakul *et al.* 1999 [13] | - Patient self-selected treatment supervisor if randomly allocated to the DOT (as opposed to SAT) arm of the study. *Comment: No information as to whether there was any input from health professionals or others on this choice.* | - Investigators at study sites were not blinded to allocation. - Outcomes determined by staff at central study unit, treatment cards contained allocation information (DOT or SAT). *Comment: information on case management included on these cards - possibly information on whether patient CB, clinic or family-supervised DOT.* - Data from study sites in year prior to study collected (routine monitoring data). Marked increase for both DOT and SAT arms during the study relative to treatment outcome data from the preceding year. | - Patients not informed about the study and the existence of other treatment options. - As an RCT this was a study with deficiencies in allocation concealment and blinding of assessors, but otherwise adequate. However, that the allocation to CB DOT and clinic DOT was not random does increase risk of selection bias for this study. - For each of the included studies, blinding of outcome assessors to DOT allocation would have been very difficult to achieve. |
| Kironde & Meintjies 2002 [26] | - Patients are normally given the option of clinic or CB DOT and told to make a choice between these two with the advice of the clinic nurse. *Comment; What advice does the clinic nurse give? No criteria stated to guide allocation.* - CB DOT supporter lives within a ‘reasonable proximity’ of the patient, patient goes to the provider. *Comment; what is considered reasonable, does greater distance affect outcomes?* | - Interviewers included DOT supervisors. *Comment; interviewers not blinded to allocation.* - Patients with missing data were excluded. *Comment; could this mean loss to follow-up is under-estimated, even though these data are not broken down for supervision type default is reported as 18.7% overall.* | - Study areas recruited randomly from 45 PHC facilities (secondary and tertiary units excluded, random selection from an SPSS algorithm - this seems adequate). - For new patients, rural residence was associated with a higher chance of treatment success. - Multiple sociodemographic factors considered in analysis – allocation of treatment supervisor was one of these, others included age, sex, education level, whether living on a disability pension etc. - Prospective study, thus more controlled than a retrospective study. |
| Miti *et al.* 2003 [27] | - Twapia (control) and Chipulukusu (intervention) compounds; “both communities are economically deprived, with poor living conditions, and both communities are served by a small health centre and have primary schools” *Comment; no quantitative data provided in the paper to substantiate comparability.* - In Twapia (control) ratio of male:female was close to 1:1 while it was 1:2 in Chipulukusu (internvention). *Comment:* *no specific mention in the paper whether this difference impacted on results.* - Initially higher patient numbers in Twapia (control) relative to Chipulukusu (intervention). *Comments: authors attribute to community adjustment to programmatic change as TB services in intervention area newly directed through HIV Home Care Programme. Unclear if this affected overall result.* - Rate of HIV co-infection in CB DOT and clinic DOT populations not specified or whether there was an overall difference between the two. Death rates are not significantly different (22% for CB DOT versus 19% for clinic DOT). - Last section of discussion mentions that: “Few tuberculosis patients in the Chipulukusu township who were not previously registered with the HCP [home care programme] opted for the daily visit by community home care volunteers. Instead, many opted for daily visits to the health centre to receive their daily medications”. *Comment: Not specified what the patient number was opting for clinic DOT and this is not mentioned as an option in the study methodology. Results for CB DOT area essentially presented as an intention-to-treat with CB DOT by the TB programme and investigators.* | - Programmatic change. *Comment; risk of reporting bias, existing program, research as a result of invitation to join a ‘Community TB care in Africa’ project.* - Training applied to volunteers from intervention area (3 days) and to nurses from both areas. *Comment; duration of nurse training and content not elaborated upon.* - No information on whether people analysing data were blinded to allocation. | - Prospective trial, thus more controlled than a retrospective study. For example, a new sputum transport system was introduced but was provided to both intervention and control arm - Limited sociodemographic commentary but age for both groups very similar (intervention: mean age of 32.4 years in intervention area and 32.5 years in control area). |
| Niazi and Al-Delaimi 2003 [28] | - Allocation to one group or the other by TB coordinator according to order of attendance *Comment: potential risk of modifying allocation.* | - Socio-demographic reporting by NGO volunteers, while outcome and compliance by TB coordinator. *Comment: prefer to have same person report or cross-checking of data extraction.* - No information on blinding to treatment allocation during data entry and analysis. - Some inaccuracies in the paper, for example stating that 83% cure rate exceeds the 85% standard. | - Specific DOT training given to NGO volunteers, though no mention if training provided to nurses or not. - Did not measure other factors that may impact on compliance such as time to reach the health facility, waiting time, satisfaction with patient care received. - Authors report that no sociodemographic factors influenced results. - Appropriate randomisation (as opposed to sequential allocation) would be helpful in strengthening results. Note that authors state in their discussion, “..because patients were randomly assigned to the 2 groups…”, this is not the case. |
| Nirupa *et al.* 2004 [31] | - Supervisor selected by doctors. *Comment; no criteria stated to guide allocation* | - Not intention-to-treat (phone call at 2 months to establish who was supervising therapy). - Information gaps (couldn't contact 12% of patients by phone). - Data computerized and cross-checked. *Comment; no information on blinding to treatment allocation during data entry and analysis.* | - Patients assigned to different treatment groups had similar sociodemographic factors (based on univariate analysis reported). - Treatment results only provided for new sputum smear-positive patients (assists with comparability). - Retrospective data and thus less robust than a prospective study. |
| Singh *et al.* 2004 [29] | - “Before starting treatment, patients residing or working in areas close to a CV were given the option of receiving DOT from that CV”. *Comment: unclear if location of CVs introduced a selection bias for the CB DOT arm.* | - Senior treatment supervisor visited community volunteers (CVs) weekly, every 2-3 days if issues and doctor visited once a month. No information given on supervision of government health workers. - No information on blinding of those evaluating data to allocation type. | - 2-day training course for CVs, though not mentioned if additional training provided to government health workers (or if this was required). - Retrospective data and thus less robust than a prospective study. |
| Tripathy *et al.* 2013 [30] | - “The patient chose the type of DOT provider in consultation with the medical officer”. *Comment: no criteria stated to guide allocation.* | - Investigators not blinded to supervisor type. - Double entry of data mentioned in the method *Comment; not sure if by different people.* - No mention of blinding to treatment allocation during data entry and analysis. | - All DOT providers (CB DOT and clinic DOT) receive training. - Retrospective data and thus less robust than a prospective study. |

**GRADE criteria assessment for included studies as relevant to comparison between CB DOT and clinic DOT.**

| **Study [ref.]** | **Starting quality based on study type ^1^** | **Study limitations (risk of bias) ^2^** | **Inconsistency of results (if consistent this does not increase quality)** | **Indirectness of evidence** | **Imprecision ^3^** | **Reporting bias** | **Overall quality of evidence** |
| --- | --- | --- | --- | --- | --- | --- | --- |
| Kamolratanakul *et al.* 1999 [13] | Low | Possible effect on primary or secondary outcome. | All studies suggest either similar outcomes between CB DOT and clinic DOT, or better outcomes from CB DOT relative to clinic DOT. Thus, there is a consistent result of at least similar treatment success from CB DOT relative to clinic DOT. However, there is substantial inter-study heterogeneity for the primary meta-analysis (84%). Even so, no studies were downgraded in quality based on inconsistency of results.  For secondary outcome, results for included studies were inconsistent: some showing greater loss to follow-up from clinic DOT, some equivalence and one study greater loss to follow-up from CB DOT (see Figure 4 on main paper and additional file 4 for further discussion of this result). | This trial compares DOT to SAT. However, specific results and discussion of different DOT providers (family member, health worker, community member) is provided, thus considered as direct evidence and not a quality lowering issue. | Imprecise (95% CI 0.13-2.39 for odd ratio (OR)) for primary outcome. Imprecise (95% CI 0.46-36.55 for OR) for secondary outcome. | For all studies, non-blinding of assessors to treatment allocation (or insufficient information on blinding) introduces a possibility of reporting bias for both the primary and the secondary outcome. Blinding of outcome assessors to DOT type is very difficult or impossible to achieve for these studies. This risk has been combined with the study limitations assessment when considering whether to downgrade study quality due to bias. As there are less than ten studies in this meta-analysis, funnel plot asymmetry tests are not appropriate. | Very low for primary and secondary outcome (downgraded due to study limitations and imprecision). |
| Kironde & Meintjies 2002 [26] | Low | Possible effect on primary outcome, secondary outcome not assessed for this study. |  | Not a quality lowering issue. | Imprecise (95% CI 0.81-1.75 for OR) for primary outcome. |  | Very low for primary outcome (downgraded due to study limitations and imprecision).  Secondary outcome not assessed |
| Lwilla *et al.* 2003 [20] | High | Possible effect on primary and secondary outcome. |  | Not a quality lowering issue. | Imprecise (95% CI 0.82-1.65 for OR) for primary outcome. Precise (95% CI 1.39-2.96 for OR) for secondary outcome, though not entirely clear which losses are due to programme and which due to study difficulties. |  | Low for primary and secondary outcome (downgraded due to study limitations and for primary outcome for imprecision). |
| Miti *et al.* 2003 [27] | Low | Possible effect on primary and secondary outcome. |  | Not a quality lowering issue. | Imprecise (95% CI 0.88-3.05 for OR) for primary outcome. Precise (95% CI 0.12-0.80 for OR) for secondary outcome. |  | Very low for primary and secondary outcome (downgraded due to study limitations and for primary outcome for imprecision). |
| Niazi and Al-Delaimi. 2003 [28] | Low | Possible effect on primary and secondary outcome. |  | Not a quality lowering issue. | Precise (same result at either end of CI, 95% CI 1.13-4.89 for OR) for primary outcome. Imprecise (95% CI 0.43-2.92 for OR) for secondary outcome. |  | Very low for primary and secondary outcome (downgraded due to study limitations and for secondary outcome for imprecision). |
| Nirupa *et al.* 2004 [31] | Low | Possible effect on primary and secondary outcome. |  | Not a quality lowering issue. | Imprecise (95% CI 0.92-1.92 for OR) for primary outcome. Precise (95% CI 0.51-0.92) for secondary outcome. |  | Very low for primary and secondary outcome (downgraded due to study limitations and for primary outcome for imprecision). |
| Singh *et al.* 2004 [29] | Low | Possible effect on primary and secondary outcome. |  | Not a quality lowering issue. | Imprecise (95% CI 0.67-1.66 for OR) for primary outcome. Imprecise (95% CI 0.61-1.72 for OR) for secondary outcome. |  | Very low for primary and secondary outcome (downgraded due to study limitations and imprecision). |
| Tripathy *et al.* 2013 [30] | Low | Possible effect on primary and secondary outcome. |  | Not a quality lowering issue. | Precise (95% CI 3.13-6.58 for OR) for primary outcome. Precise (95% CI 0.19-0.63 for OR) for secondary outcome. |  | Low for primary and secondary outcome (study limitations downgrade evidence but large effect size means that evidence is of overall low quality). |

^1^ RCTs start as high quality, observational studies start as low quality and this has been applied to the non-randomised studies, even if interventional rather than observational.

^2^ In spite of existing study limitations, the authors of this systematic review have chosen to include all included studies in the meta-analysis as the selection criteria for the systematic review meant that these included studies had the least risk of bias of all of the studies assessed.

^3^ Precision judgment based on whether top and bottom of confidence interval were on the same or different sides of the ‘1’ value.
